# Chromosome clustering in mitosis by the nuclear protein Ki-67

**DOI:** 10.1042/BST20210717

**Published:** 2021-11-16

**Authors:** Konstantinos Stamatiou, Paola Vagnarelli

**Affiliations:** College of Medicine, Health and Life Science, Brunel University London, Centre for Genomic Engineering and Maintenance (CenGem), London UB8 3PH, U.K.

**Keywords:** chromosomes, Ki-67, mitosis

## Abstract

Ki-67 is highly expressed in proliferating cells, a characteristic that made the protein a very important proliferation marker widely used in the clinic. However, the molecular functions and properties of Ki-67 remained quite obscure for a long time. Only recently important discoveries have shed some light on its function and shown that Ki-67 has a major role in the formation of mitotic chromosome periphery compartment, it is associated with protein phosphatase one (PP1) and regulates chromatin function in interphase and mitosis. In this review, we discuss the role of Ki-67 during cell division. Specifically, we focus on the importance of Ki-67 in chromosome individualisation at mitotic entry (prometaphase) and its contribution to chromosome clustering and nuclear remodelling during mitotic exit.

## Introduction

**Figure 1.**
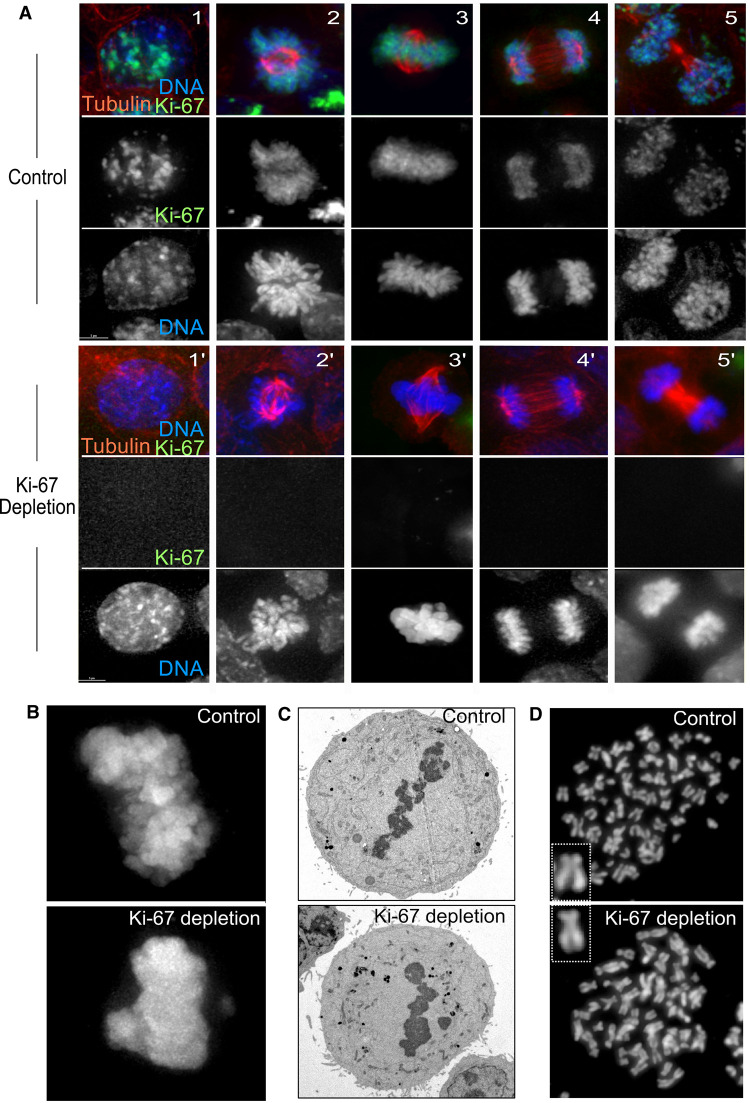
Ki-67 depletion leads to abnormal chromosome clustering in metaphase. (**A**) Interphase and mitotic stages of HCT116 cells in the presence (Control) or absence (Ki-67 depletion) of Ki-67. Ki-67 in green, tubulin in red and DNA in blue. The panels from left to right present an interphase nucleus (1, 1′), prometaphase (2, 2′), metaphase (3, 3′), anaphase (4, 4′) and telophase (5, 5′). (**B**) Metaphase chromosomes of HeLa cells with (top panel — Control) or without (bottom panel — Ki-67 depletion) Ki-67 after paraformaldehyde fixation. (**C**) Electron microscopy images of metaphase chromosomes from HeLa cells with (top panel — Control) or without (bottom panel — Ki-67 depletion) Ki-67. Images are courtesy of Daniel Booth, Nottingham. (**D**) Metaphase chromosome spreads of nocodazole arrested HeLa cells with (top panel — Control) or without (bottom panel — Ki-67 depletion) Ki-67.

Multicellular organisms are constituted by cells with different functions resulting from the specific gene expression programs characteristic for each cell type that need to be maintained from one generation to the next. A challenge to the faithful transmission of this information is represented by mitosis. In mitosis, the genome of proliferating cells undergoes remarkable series of structural changes at mitotic entry that lead to chromosome condensation and the formation of mitotic chromosomes [1,2]. Mitosis begins with prophase: at this stage the condensin complexes drive chromosome condensation that continues until metaphase. During prophase, the formation of the mitotic spindle also begins with the two pairs of centrioles moving to opposite poles and microtubules polymerising from the duplicated centrosomes [3]; at the transition between prophase and prometaphase, the nuclear envelope break-down represents an essential step for spindle assembly [4]. In prometaphase, microtubule dynamics (rapidly growing and shrinking microtubules) lead to the capture of chromosomes at the kinetochores and the assembly of a bi-polar spindle begins (Figure 1A, panel 2). As prometaphase progresses, the chromosomes are pulled in opposite directions by microtubules connected to opposite spindle poles, thus leading to chromosome oscillations, until the pole-directed forces are balanced [5]. Sister chromatids do not break apart during this process because they are firmly held together by cohesin molecules present at the centromeres. In metaphase (Figure 1A panel 3) all centromeres of all the chromosomes are aligned at the metaphase plate (spindle equator) and the chromosomes come in tight contact with each other in a very small volume; however, the chromosomes maintain their individuality and do not collapse in a single mass (mitotic clustering). The progression of cells from metaphase into anaphase is defined by the separation of sister chromatids when the protease separase cleaves the cohesin rings. During the first part of anaphase, the kinetochore microtubules shorten, and the chromosomes move toward the spindle poles (anaphase A) [6], followed by the spindle poles separation (anaphase B) [7]. At the end of this mitotic stage, the chromosomes reach their maximum compaction and cluster together (Figure 1A, panel 4). Mitosis ends with telophase, where the nuclear membrane reforms, and the chromosomes begin to decondense into their interphase conformations [8] (Figure 1A, panel 5). At the end, cytokinesis occurs when the division of the cytoplasm into two daughter cells takes place. Mitosis is a very well-regulated event and defects in this stage of the cell cycle can lead to harmful consequences for the viability of proliferating cells. Alterations during mitotic processes can result in chromosome mis-segregation, genomic instability and eventually transcription reprogramming, a main characteristic of cancer.

Being so critical for cell survival, this process is highly controlled not only biochemically but also topologically. The position of chromosomes, spindle and cleavage furrow are important cues for an error-free mitosis. Microscopy analysis of chromosome behaviour during cell division has fascinated many scholars starting from Boveri [9]. As microscopy techniques have improved the visualisation capacity and molecular biology has enhanced our understanding of how chromatin is folded, we can start building a high-definition picture of the drivers and the power of this interesting chromosome gymnastics during cell division.

While chromosomes condense in mitosis, major changes in their mobility occur; these are driven by the very dynamic microtubules and spindle forces. The chromosomes will need to form a tight metaphase plate whilst maintaining their individuality, thus allowing for a swift separation of sister chromatids at the onset of anaphase without causing entanglements. When the migration of sister chromatids is completed, the chromosomes will reach their maximum compaction [10,11] and maintain tight contacts with each other: this will facilitate the reformation of a nuclear membrane that entraps the entire genome. Failure in these mechanisms will lead to the formation of abnormal nuclei and micronuclei, a hallmark of many cancers.

The molecular effectors that are in place to maintain the chromosomes separated in the first part of mitosis (a) and to bring them together at the end (b) are starting to emerge. Surprisingly (or not), recent evidence seems to suggest that the same molecule is involved in both aspects: Ki-67.

In this review, we are going to provide the reader with the key aspects of this process, the major findings and we will highlight the main open questions.

## Ki-67 and the perichromosomal layer

To understand the molecular mechanisms that allow chromosomes to maintain their individuality (inhibit clustering) in early mitosis but then to be able to coalesce (favour clustering) at the end, we need to understand the outermost part of the mitotic chromosomes: the perichromosomal layer. The mitotic chromosome is composed of a chromosome scaffold fraction, the centromere/kinetochore, telomeres and a sheath of proteins surrounding each chromosome defined as the chromosome periphery or perichromosomal layer [2]. During the years these chromosome compartments have been studied extensively, except for the chromosome periphery which is the least characterised compartment. The chromosome periphery exists at the outer surfaces of individual chromosomes [12–15] and constitutes approximately one third of the protein mass of mitotic chromosomes [2,16]. This protein layer appears on chromosomes at prometaphase and disappears at telophase when the nuclear envelope reforms [17], although there are variations in the timing of localisation of each individual protein component. Ki-67 is one of the earliest proteins to associate with the chromosome periphery [18] and acts as a scaffold for the formation of the chromosome periphery [15].

**Figure 2.**
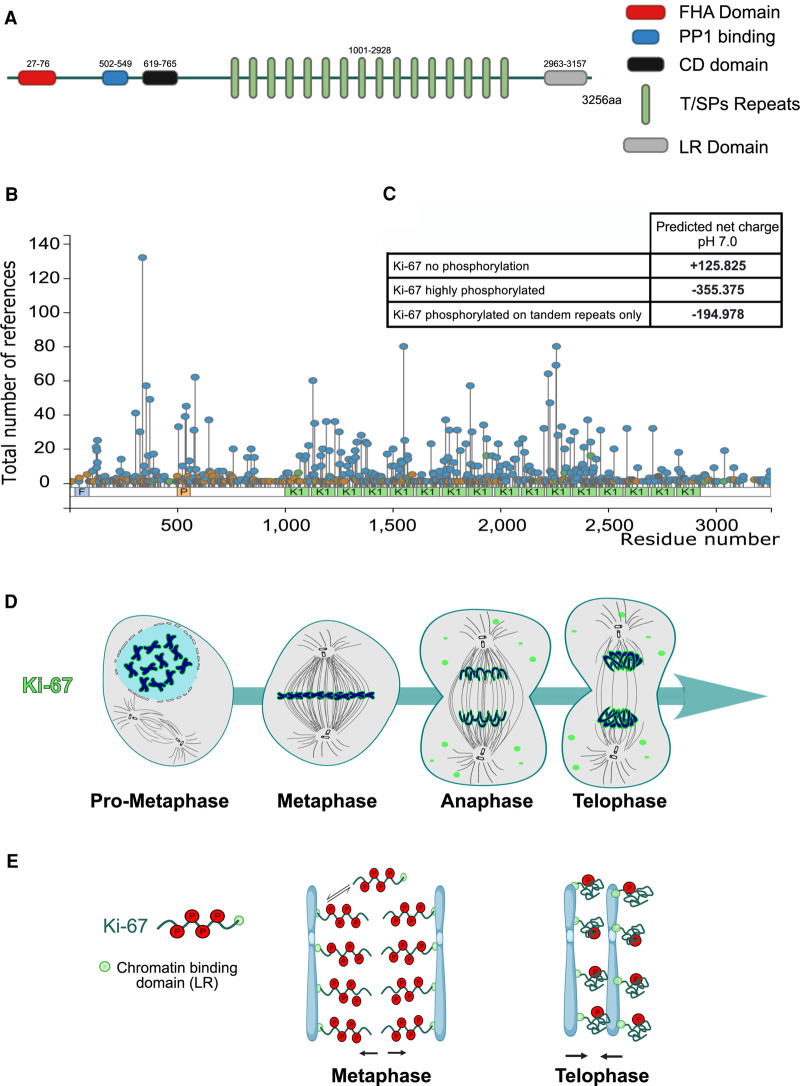
A charge-dynamic model for Ki-67 function during mitosis. (**A**) Schematic diagram of Ki-67 and its domains. The numbers indicate the amino acids. (**B**) Lollypop graph representing all the known phosphorylation sites (blue), the tandem repeats (K1: green), the FHA domain (F: light blue) and the PP1 binding site (P: orange) (from https://www.phosphosite.org). (**C**) Table indicating the net charge of Ki-67 when de-phosphorylated or phosphorylated. (**D**) Diagram showing the localisation of Ki-67 (green) during mitotic progression. (**E**) Scheme representing a possible model for a repulsive function of Ki-67(green) in early mitosis and a cohesive function in late mitosis. Phosphorylations are represented by the red circles.

Ki-67 for many years has been used as a prognostic marker for multiple types of cancer [19–23], as it is expressed only in proliferating cells but is down-regulated in resting G0 cells [19]. Despite its usefulness in clinics, much less attention has been paid to the molecular functions of Ki-67 that, for decades, remained largely unknown. Recent studies associated Ki-67 with cell cycle regulation, heterochromatin maintenance and the assembly of the perichromosomal layer on mitotic chromosomes [16–19]. Ki-67 appears to have roles in both interphase and mitosis as its cellular distribution dramatically changes during the cell cycle [20] (Figures 1A and 2D). In interphase, it seems to be required for heterochromatin organisation [5] and for the localisation of the nucleolar organising regions [24,25]. In mitosis, Ki-67 is essential for the formation of the perichromosomal layer, and the equal segregation of several proteins associated with this compartment that will then become part of the nucleolus [18]. Recent studies have shown that the histone chaperone chromatin assembly factor-1 (CAF-1) functions as a chaperone for Ki-67 [25,26]. The p150 subunit of CAF-1 seems to regulate the localisation of Ki-67 to the chromosome periphery during mitosis and to the G1 foci (small nuclear clusters of Ki-67 in early G1) [25,26]. p150 co-localises with Ki-67 during all stages of the cell cycle and it seems that the regulation of Ki-67 depends on the SUMOylation interacting motif within p150 [25,26]. These findings may partly explain how Ki-67 is recruited to the chromosome periphery.

The mitotic chromosome periphery consists of numerous proteins with diverse functions during the cell cycle. The number of components is still growing as more proteins associated with the chromosome periphery are being identified by proteomic screens of isolated chromosomes [27,28]. It is unclear yet whether the chromosome periphery is a singular domain or represents the assembly of multiple, partially overlapping domains (subcomplexes) which somehow collaborate to control the different roles of this structure. Correlative light and serial block-face scanning electron microscopy experiments strongly suggested that depletion of Ki-67 results in the loss of most, if not all, of the periphery compartment [18]. These findings, bring us to the conclusion that Ki-67 is a master regulator for the formation of the chromosome periphery and raise many hypotheses for the possible roles of this chromosome compartment.

Among the components of the chromosome periphery there are several ribonucleoproteins and RNAs [2,13,28,29]. Ki-67 regulates the formation of the chromosome periphery [18] since depletion of Ki-67 in human cells led to the dispersal of all the chromosome periphery components tested, including the nucleolar proteins nucleolin, nucleophosmin/B23, NIFK, PES1, cPERP-B, cPERP-C, cPERP-D, cPERP-F and pre-ribosomal RNAs; however, the depletion of these components did not alter the perichromosomal localisation of Ki-67 [18,22,30]. The depletion of different components of the chromosome periphery leads to slightly different phenotypes that can be classified in different subgroups: (a) the presence of mis-aligned chromosomes and spindle abnormalities, (b) presence or absence of micronuclei, (c) increased apoptosis, (d) formation of a single nucleolus: these phenotypes could suggest the existence of different networks with specific functions within the layer. For example, nucleolin and B23, proteins of the chromosome periphery, are interacting with each other [31] and both have a role in mitotic spindle formation and in the maintenance of the stability of kinetochore-microtubule attachments for faithful chromosome segregation [31–32]. Nucleolin depletion leads to chromosome misalignment, defects in mitotic spindle formation and apoptosis; B23 depletion follows the same phenotypic pattern [31–33]. Nucleolin seems to be upstream of B23 and regulates its localisation at the chromosome periphery [22,31]. Both, nucleolin and B23 are important for kinetochore-microtubule attachments with their depletion leading to monotelic or syntelic kinetochore-microtubule attachments [31]; B23 has also been shown to have a role in centrosome duplication [34,35]. In this respect, Ki-67 seems to be upstream of all the events leading to the formation of the chromosome periphery. These findings indicate that there is a hierarchy and possibly a clustering of functions within the components of the chromosome periphery; however, there are many other components with functions which are not yet known, highlighting the need for further studies to map the entire ‘peripherome’ and understanding its functions.

## Function of the perichromosomal layer in early and late mitosis

The perichromosomal layer appears in prophase and it is dismantled at the end of cell division when the nuclear envelope is fully reformed. For identifying the possible functions of Ki-67 through the cells cycle, a human Ki-67-mAID-mClover cell line (HCT116) that allows endogenous Ki-67 to be both visualised (via mClover) and targeted for rapid proteasomal degradation (via the mAID degron) was generated [36]. The rapid removal of Ki-67 after mitotic entry, resulted in mitotic chromosomes disorganisation which became shorter and thicker (swollen) in comparison with the control cells [36]. Further experiments revealed that Ki-67 depletion resulted in the mislocalisation of both TopoIIα and the condensin II complex member hCAP-H2, suggesting possibly a link between chromosome periphery proteins and chromosome structure proteins [36]. In addition, double depletion of Ki-67 and SMC2 (a core subunit of both condensin complexes) led to the formation of ball-like chromosome clusters with no sign of discernible thread-like structures [37]. Taking all together, it seems that Ki-67 and condensins have independent yet cooperative functions in supporting the structural integrity of mitotic chromosomes. Other studies have shown no obvious defects in chromosome spreads obtained from depletion [18] (Figure 1D) or Knock out [38] of Ki-67 in human cells. Moreover, using an assay designed to test the intrinsic architecture of metaphase chromosomes, where chromosomes are induced to unfold by removal of divalent cations and then induced to re-fold by addition of Mg^2+^ [39], no clear difference between control and Ki-67-depleted chromosomes were observed [18].

Nevertheless, all the studies are in agreement with the fact that mitotic chromosomes in cells lacking Ki-67 are abnormally clumped together [16,18,38], a phenotype particularly evident when cells are aligned at the metaphase plate (Figure 1A, panel 3′ and Figure 1B). Individual chromosomes are no longer distinguishable either by light (Figure 1B) or electron microscopy Figure 1C). This phenotype is observed when analysing the cells either by paraformaldehyde fixation or live-cell imaging and it is not a consequence of an abnormal chromosome structure (as discussed above) since metaphase spreads analyses showed normal chromosomes (Figure 1D). In line with these observations, we could envisage that the 87–150 nm thick chromosome periphery [16,38] covering the entire outer surface of chromosomes could possibly act to keep the chromosomes separate and prevent chromosomes from collapsing into a single chromatin mass at the crowded metaphase plate. In a study that used FRET biosensors to monitor mitotic phosphorylation and RNAi screening platforms, it was suggested that Ki-67 was the only candidate shown to contribute to the spatial separation of mitotic chromosomes [38]. The establishment of a dual tagging system by fusing fluorescent reporters to both the C and N termini of Ki-67, led to the proposal that Ki-67 has a brush-like arrangement around the chromosomes, characteristic of polymeric surfactants, and extends out 87 nm from an anchor point at the chromosome into the cytoplasm [38] (Figure 2E, metaphase). Further characterisations however suggested that during prophase, Ki-67 is not required for the initial individualisation of chromosomes as they condense [38].

Ki-67 depleted chromosomes are more prone to stick to each other and behave like a single contiguous and relatively immobile mass (Figure 1A, panel 3′, 1B,C), thus resulting in impaired spindle assembly and metaphase plate formation, which prolonged the progression from prometaphase to anaphase (mitotic delay) [38]. Nevertheless, human [38] and mouse Ki-67 [38] knock-out cells can be generated indicating that cell cycle progression can still occur even in the absence of Ki-67. However, it still needs to be established if, in these knock-out systems, compensatory mechanisms are triggered in order to sustain cell division without Ki-67.

These results raised the hypothesis that Ki-67 acts as a steric and electrostatic coating on chromosomes by forming a sort of biological surfactant important to maintain the individuality of each mitotic chromosome [38]. Indeed, the transient expression of Ki-67 truncation mutants (different lengths of the peptide chain) in Ki-67 depleted cells rescued the phenotype. The phenotype rescue efficiency correlated with the protein size and predicted net charge of the different constructs, suggesting that the size and overall electric charge might be important for Ki-67 ability to act as a biological surfactant [38]. Interestingly, high-level overexpression of core histones, and most specific H2B, in Ki-67 depleted cells restored chromosome separation in prometaphase (not to the same extent as the full length of Ki-67 protein), possibly through the addition of positive electrical charges [38]. However, this interpretation is difficult to envisage since the addition of positive electrical charges around the mitotic chromosomes caused by the overexpression of histone proteins implies the ability of incorporating additional molecules of H2B into the DNA by the formation of extra nucleosomes that apparently could lead to increased DNA condensation; this hypothesis has not yet been proven and it does not seem that Ki-67 depleted chromosomes are more weakly compacted as shown by micrococcal nuclease digestions [38]. A similar rescue of chromosome individualisation was obtained upon treatment with trichostatin A, a histone deacetylase inhibitor, [40]. This could suggest that maybe chromatin organisation or epigenetic modifications could also be contributing to the phenotype.

As mentioned before, the maximum chromosome compaction is achieved at the end of mitosis when the chromosomes are tightly clustered before they will start decondensing for re-entering a new cell cycle. A recent study has also implicated Ki-67 at this stage with an apparently opposite effect to the one exerted in early mitosis [40]. At mitotic exit, the brush-like arrangement forming a repulsive barrier during the early stages of mitosis [38] collapses, thus inactivating the surfactant function of the protein and promoting chromosome clustering (Figure 2E, telophase) [40]. Indeed, by using the dual tagging system it was shown that, in prometaphase, Ki-67 N-terminus localised 66 ± 27 nm towards the cytoplasm relative to its C-terminus, while this distance was decreased to 32 ± 32 nm after induced chromosome clustering by flavopiridol (a drug that inhibits CDK1 and triggers a biochemical mitotic exit) [41], suggesting that Ki-67 extended molecular brushes collapse when chromosomes cluster during mitotic exit [40]. After further investigation, it was observed that cells overexpressing H2B-mNeonGreen, at levels that are sufficient to prevent chromosome separation in prometaphase, still failed to cluster chromosomes upon mitotic exit in the absence of Ki-67 [39,40]. Notably, chromosome clustering was restored only after the transient expression of EGFP-Ki-67 [40]. One of the possibilities to explain this change of function between early and late mitosis could be that it is driven by the recruitment of protein phosphatase 1 (PP1). PP1 can bind to Ki-67 in late mitosis but not early mitosis [18]. However, a PP1 binding motif mutant, that can abolish PP1 binding, showed that chromosomes were still able to cluster normally [40]. These results led to the conclusion that Ki-67 promotes chromosome clustering through its intrinsic properties rather than through the recruitment of PP1 [40]. Chromosome clustering can also be achieved artificially by co-expressing H2B-fused to either FRB or FKBP domains: the addition of rapamycin leads to stabilisation of contacts between histones and promotes chromosome clustering within a few minutes, suggesting that increasing chromosome adhesion is sufficient to induce chromosome clustering, even in the absence of Ki-67 [40].

## How can the same molecule have such opposite effects?

The central region of Ki-67 consists of 16 tandem repeats that are encoded by a single large exon (6845 bp) (Figure 2 A) [42,43]. Studies on the function of Ki-67 revealed that the repeats are important for the correct localiation of Ki-67 at the chromosome periphery and that the efficient chromosome periphery targeting of Ki-67 depends on the number of these repeating units [38,44]. However, the FHA domain, the leucine/arginine-rich (LR) C-terminal chromatin-binding domain and the phosphorylation status of the protein, are all important for the localisation of Ki-67 during mitosis [42,44–47]. The central region of Ki-67 also contains residues phosphorylated by CDK1 (Figure 2B) [42,44–47]. In interphase, Ki-67 is dephosphorylated and forms immobile fibre like structures around the nucleoli, and it is associated with the nucleolar heterochromatin [46–48]. At the transition from G2 into mitosis, Ki-67 is hyperphosphorylated by CDK1 and this phosphorylation shift causes Ki-67 to weaken its affinity for the DNA and become highly mobile. Notably, fluorescence recovery after photobleaching (FRAP) experiments showed that GFP-pKi-67 was rapidly exchanging from the chromosome periphery before anaphase onset (*t*_1/2_ recovery time of 11.82 ± 3.85 s in metaphase) while the recovery rate for GFP-Ki-67 was considerably decreased after the onset of anaphase (with a *t*_1/2_ recovery time were 45.30 ± 14.55 s in anaphase and 48.79 ± 10.97 s in telophase) [49]. At anaphase, Ki-67 interacts with PP1γ [6,18,24,44] and it is dephosphorylated [49]. Interestingly, the use of staurosporine, a general kinases inhibitor, in metaphase arrested cells, resulted in a partial disassociation of Ki-67 from the chromosome periphery and abnormal cytoplasmic foci formation, indicating that the phosphorylation of Ki-67 during mitosis has a regulatory function for its localisation [45]. These results indicate that Ki-67 is regulated by phospho-switches during cell cycle that, in turn, control the kinetics and the ability to interact with higher-order chromatin structures.

Recently, published data have shed some light on the importance of chromosome clustering at the end of mitosis. In fact, cells lacking Ki-67 displayed nuclei contaminated with cytoplasmic components, including mature ribosomes [40], thus demonstrating that the end-of-mitosis clustering contributes to establish the nuclear–cytoplasmic compartmentalisation necessary for the new G1 cells. Small molecules could still be exported by the nucleus–cytoplasmic trafficking, but big assembled components could not and therefore must be excluded from the resealed nuclear envelope.

This also opens another interesting possibility regarding the function of the perichromosomal layer. In fact, this compartment could represent a vehicle to mediate a quick start re-assembly of some nuclear sub-compartments. This idea is also supported by the observation that B23 and nucleolin (both nucleolar proteins in interphase and associated with the chromosome periphery in mitosis) were unevenly distributed in Ki-67-depleted daughter cells at cytokinesis [18]. Moreover, rRNA transcription appears less efficient in Ki-67-depleted cells [18,50]. Ki-67 seems to delay the early nucleolar pre-rRNA cleavage hierarchy; however, other data do not support a role of Ki-67 in rRNA transcription. Interestingly, loss of some pre-rRNAs form the chromosome periphery surface has been shown to lead to the mislocalisation of other periphery components, including fibrillarin [51]. Considering that Ki-67 has a brush-like arrangement, the outer (N-terminal) part of the Ki-67 brush contains both the phosphopeptide binding FHA domain and the PP1-binding domain suggesting that Ki-67 could allow PP1 to explore the surface of mitotic chromosomes, binding phospho-proteins, and leading to the efficient dephosphorylation of diverse nucleolar proteins in the perichromosomal layer, thus helping to promote efficient nucleolar reassembly and reactivation [2].

## Outlook and future directions

Looking at the models so far available for the chromosome individualisation and clustering carried out by Ki-67 in early and late mitosis respectively, we are still missing some key molecular explanations. The hypothesis considers that the positive charges of the protein in early mitosis are responsible for the repulsive effect between chromosomes in the early stages; however, this model does not take into account the protein post-translational modifications and its dynamics. In fact, if we factor in the phosphorylations that occur in early mitosis, the Ki-67 net charge shifts from +125.85 to −355.375 (for a full phosphorylation) or −194.978 (for the phosphorylation of the repeats only) (Figure 2C,D). This may well explain why Ki-67 is very dynamic in early mitosis as the net charges will not favour DNA binding. Moreover, being Ki-67 highly phosphorylated in the middle region during mitosis, the negative charges will repulse each other and possibly contribute to the extended form of the molecule observed by FRET analyses (Figure 2E, metaphase). Later in mitosis, the dephosphorylation leads to a major change in charges that will increase the affinity for DNA and, possibly, alter its structure, allowing a higher degree of folding no longer prevented by the early mitotic phosphorylation (Figure 2E, telophase). In this case, Ki-67 will be highly positively charged and being at the periphery could have high affinity for both the DNA of the chromosome where it is located on, and also for the DNA from other nearby chromosomes, thus leading to clustering (Figure 1E, telophase). This model implies that some critical phosphorylation sites at the C-terminus have a major effect on the modulation of Ki-67 binding to DNA. Alternatively, the significant change in charge between early mitosis and mitotic exit could alter both the surfactant properties of the molecule and its dynamics. Possibly, the extended structure (in early mitosis) is more mixable than the compact structure at the end of mitosis, thus leading to a change in its dynamic behaviour and clustering/resident time around the chromosomes. These hypotheses are not mutually exclusive, and both can contribute to overall behaviour of Ki-67 during mitotic progression. In fact, the C-terminus alone has DNA binding abilities *in vitro* ([45] and our unpublished results) and it is necessary for binding to the mitotic chromosomes [38,49] but it is not enriched at the chromosome periphery, thus suggesting that other properties of the molecule (either presence of interaction domains with other proteins or critical biophysical properties) contribute to the peripheral localisation. The enrichment at the chromosome periphery seems to be provided by the extended Ki-67 central repeats (only when most of the repeats are present) [38]; indicating that possibly the de-mixing properties of the molecules are also important. This is an essential aspect to resolve in order to fully understand the biology of Ki-67 and more investigations are needed to nail down the exact mechanism. Therefore, from a molecular point of view, we still do not have a clear picture on how the collapsing of mitotic chromosomes occur in the absence of Ki-67 and more detailed analyses of the protein structure, subcomplexes of the perichromosomal layer and chromatin organisation need to be investigated to fully understand these important aspects of the mitotic chromosomes.

We still do not know exactly what the biological function is of maintaining chromosomes separated from each other in early mitosis. A possible hypothesis could be that if chromosomes cluster prematurely, the spindle could not easily capture the kinetochores thus leading to spindle structural defects or mitotic delay as some studies seem to suggest [7,22,38,52,53]. However, other studies have reported normal proliferation in cells depleted of Ki-67 [54]. Regardless, Ki-67 knock-out mice and cell lines are viable [22,38] indicating that cells can adapt to the loss of the protein and overcome these consequences, even if the system seems to be less resilient to stress [55,56].

The evidence that cells and organisms can survive without a protein that seems to have such important functions in mitosis it is quite interesting and puzzling at the same time but clearly suggests that future work is needed to clarify if adaptation mechanisms are in place to bypass Ki-67 absence or if safe-valve mechanisms exist that take over when one is failing.

## Perspectives

- *Highlight the importance of the field.* Faithful chromosome segregation requires precise and coordinated chromosome movements during the different stages of mitosis; chromosomes must congress to the metaphase plate but still maintain separation in early mitosis while their clustering at the later stages of mitosis allows for a correct reformation of the nuclear envelope and the establishment of a nuclear/cytoplasmic compartmentalisation.
- *A summary of the current thinking*. Recent studies have highlighted the important role of the chromosome periphery in this process and, in particular the pivotal function of Ki-67, a well-known proliferation marker.
- *A comment on future directions*. The identification of the molecular mechanisms for the perichromosomal layer structure and function and how Ki-67 can direct almost opposite roles at different stages of mitosis will provide further important details on how cell division is regulated.

## Abbreviations

CAF-1: chaperone chromatin assembly factor-1
FRAP: fluorescence recovery after photobleaching
PP1: protein phosphatase one
